# Therapeutic effects of mitoquinol during an acute heat stress challenge in growing gilts

**DOI:** 10.1093/jas/skae250

**Published:** 2024-08-30

**Authors:** Edith J Mayorga, Alyssa D Freestone, Tori E Rudolph, Melissa Roths, Megan A Abeyta, Sonia Rodríguez-Jiménez, Brady M Goetz, Julie Opgenorth, Joshua T Selsby, Lance H Baumgard

**Affiliations:** Department of Animal Science, Iowa State University, Ames, IA 50011, USA; Department of Animal Science, Iowa State University, Ames, IA 50011, USA; Department of Animal Science, Iowa State University, Ames, IA 50011, USA; Department of Animal Science, Iowa State University, Ames, IA 50011, USA; Department of Animal Science, Iowa State University, Ames, IA 50011, USA; Department of Animal Science, Iowa State University, Ames, IA 50011, USA; Department of Animal Science, Iowa State University, Ames, IA 50011, USA; Department of Animal Science, Iowa State University, Ames, IA 50011, USA; Department of Animal Science, Iowa State University, Ames, IA 50011, USA; Department of Animal Science, Iowa State University, Ames, IA 50011, USA

**Keywords:** antioxidant, hyperthermia, MitoQ, oxidative stress

## Abstract

Study objectives were to evaluate the effects of mitoquinol (**MitoQ**) on production parameters, gastrointestinal tract (**GIT**; stomach and small and large intestines) weight, and circulating leukocytes during a 24-h acute heat stress (**HS**) challenge. Crossbred gilts [*n* = 32; 49.1 ± 2.4 kg body weight (**BW**)] were blocked by BW and randomly assigned to 1 of 4 environmental-therapeutic treatments: 1) thermoneutral (**TN**) control (*n* = 8; **TNCON**), 2) TN and MitoQ (*n* = 8; **TNMitoQ**), 3) HS control (*n* = 8; **HSCON**), or 4) HS and MitoQ (*n* = 8; **HSMitoQ**). Pigs were moved into individual pens and allowed to acclimate for 6 d. The study consisted of 2 experimental periods (**P**). During P1 (2 d), all pigs remained in TN conditions (20.6 ± 1.5 °C) and were fed ad libitum. During P2 (24 h), pigs were fed ad libitum and exposed to either TN or constant HS (37.3 ± 1.3 °C). Mitoquinol (40 mg/d) was orally administered twice daily (0700 and 1800 hours) during P1 and P2. As expected, pigs exposed to HS had increased rectal temperature, skin temperature, and respiration rate (+1.5 °C, +8.7 °C, and +86 bpm, respectively; *P* < 0.01) compared to their TN counterparts. Compared to TN, HS pigs had decreased feed intake (67%; *P* < 0.01) and significant BW loss (+1.5 vs. −1.9 kg, respectively; *P* < 0.01). Total GIT weight was decreased in HS relative to TN pigs (*P* < 0.01), and this was influenced by decreased luminal contents (2.43 vs. 3.26 kg, respectively; *P* < 0.01) and reduced empty GIT mass (3.21 vs. 3.48 kg, respectively; *P* = 0.03). Stomach contents remained similar between TN and HS pigs (*P* > 0.54) but tended to increase in MitoQ relative to CON pigs (0.90 vs. 0.63 kg, respectively; *P* = 0.08). Stomach content as a percentage of the previous 24 h feed intake was increased in HS compared to the TN controls (93% vs. 31%; *P* < 0.01). In contrast, small and large intestinal contents were decreased in HS compared to TN pigs (23% and 49%, respectively; *P* < 0.01). Liver weight decreased in HS relative to TN pigs (1.15 vs. 1.22 kg, respectively; *P* = 0.02), and was decreased in MitoQ compared to CON pigs (1.13 vs. 1.24 kg; *P* < 0.01). Circulating lymphocytes tended to be decreased in HS relative to TN pigs (16%; *P* = 0.07). In summary, acute HS increased all body temperature indices, negatively influenced animal performance, and differentially altered GIT motility as evidenced by decreased gastric emptying and increased intestinal transit. However, MitoQ supplementation did not appear to ameliorate these effects.

## Teaser

Mitoquinol, a mitochondria-targeted antioxidant, has been shown to improve growth performance in heat-stressed barrows, but the anticipated beneficial effects were not replicated in the current study using heat-stressed gilts. Further research is warranted to determine if oxidative stress is part of the mechanism responsible for the negative effects of heat stress and if alleviating oxidative stress improves production performance.

## Introduction

Heat stress (**HS**) negatively affects almost every animal agriculture sector, resulting in billions of dollars in lost profit annually and decreased production of high-quality protein for human consumption (Baumgard and Rhoads, 2013). HS-induced economic losses stem from inconsistent and suboptimal growth, reduced reproductive performance, increased morbidity and mortality, reduced carcass quality, and decreased facility efficiency (Baumgard and Rhoads, 2013; Mayorga et al., 2019). Despite continuous engineering efforts (i.e., cooling systems, barn management) to reduce its negative impacts, HS remains a continued obstacle to efficient pork production. Thus, nutritional and physiological strategies are needed to optimize pig performance during the warm summer months.

The mechanisms underlying HS-induced alterations are likely multifaceted; however, an increasing body of evidence across various species suggests intestinal hyperpermeability is at the epicenter of HS-related pathology (Lambert, 2009; Pearce et al., 2013; Gabler and Pearce, 2015; Gabler et al., 2018; Lim, 2018; Koch et al., 2019; Lian et al., 2020; Mayorga et al., 2020; Rostagno, 2020; Ortega and Szabó, 2021). One of the ostensible causes of intestinal hyperpermeability is via HS-induced oxidative stress (**OS**) within the intestinal epithelium (Hall et al., 1999; Oliver et al., 2012; Pearce et al., 2013; Yu et al., 2013; Liu et al., 2016). Oxidative stress occurs when the equilibrium between reactive oxygen species (**ROS**) production and antioxidant defenses is disrupted, resulting in damage to cellular components (Lushchak, 2014). Additionally, OS may compromise the gastrointestinal tract (**GIT**) by disrupting tight junction complexes, thereby increasing barrier permeability (Rao, 2008).

Given that OS may play a role in HS-induced intestinal damage, one strategy is to provide supplemental antioxidants (e.g., vitamin E, selenium). However, the benefits of dietary antioxidants during HS are inconsistent, as some report reduced OS (Maseko et al., 2014; Liu et al., 2016), while others do not (Liu et al., 2018a, b; Silva-Guillen et al., 2019). Additionally, several experiments supplementing traditional antioxidants during HS have shown some improvement in growth (McKee et al., 1997; Bollengier-Lee et al., 1999; Alhidary et al., 2015), but others have not (Shakeri et al., 2019; Silva-Guillen et al., 2019). Reasons for the inconsistencies are not clear, but differences in the type and quantity of antioxidants utilized and species evaluated are obvious potential explanations. Regardless, there remains a need to create an effective and consistent dietary strategy to alleviate the negative impact of OS during HS.

Mitochondria are the primary source of endogenous ROS production. Thus, mitochondria-targeted antioxidants may be more beneficial in ameliorating OS than traditional broad-acting antioxidants. Mitoquinol (**MitoQ**) is an orally available antioxidant that targets and accumulates in the mitochondria (Murphy and Smith, 2007). MitoQ has been shown to ameliorate tissue damage and improve mitochondria function during sepsis (Lowes et al., 2008), ischemia (Adlam et al., 2005), and fatty liver disease (Mercer et al., 2012). Further, we have previously reported that MitoQ ameliorated body weight (**BW**) loss and inappetence during severe heat load in growing barrows (Mayorga et al., 2024). Therefore, our objectives were to further evaluate and confirm the results from our previous MitoQ study. We hypothesized that supplementing growing gilts with MitoQ would alleviate the negative consequences of HS on production parameters during an acute HS insult.

## Materials and Methods

### Animals, housing, and experimental design

All experimental procedures followed the guidelines for the ethical and humane use of animals for research according to the Guide for the Care and Use of Agricultural Animals in Research and Teaching (FASS, 2010) and were approved by the Iowa State University Institutional Animal Care and Use Committee (IACUC-18-314).

Thirty-two crossbred gilts (49.1 ± 2.4 kg BW) were enrolled in an experiment conducted at the Iowa State University Swine Nutrition Farm facility (Ames, IA). Based on BW, pigs were allocated to 8 blocks and enrolled in a 2 × 2 factorial design and randomly assigned to 1 of 4 environmental-therapeutic treatments: 1) thermoneutral control (**TNCON**; *n* = 8), 2) TN and MitoQ (**TNMitoQ**; *n* = 8), 3) HS control (**HSCON**; *n* = 8), or 4) HS and MitoQ (**HSMitoQ**; *n* = 8). Pigs were housed in individual crates (57 × 221 cm) equipped with a stainless-steel feeder and a nipple drinker and were fed ad libitum a standard diet formulated to meet or exceed the nutritional requirements of growing pigs (NRC, 2012; Table 1). Water was provided ad libitum throughout the experiment.

**Table 1.** Ingredient composition of diet (as-fed basis)

Ingredient	%
Corn, yellow dent	78.77
Soybean meal	17.72
Soybean oil	0.50
L-Lysine	0.32
l-Threonine	0.08
dl-methionine	0.05
Monocalcium phosphate	1.09
Limestone	0.81
Salt	0.35
Phytase^1^	0.02
Vitamin–mineral premix^2^	0.29

^1^Optiphos 5000, Huvepharma, Sofia, Bulgaria.

^2^Vitamin–mineral premix provided the following (per kilogram diet): 8,400 IU/kg of vitamin A, 1,540 IU/kg of vitamin D_3_, 45 IU of vitamin E, 0.03 mg of vitamin B_12_, 2.2 mg of menadione, 4.2 mg of riboflavin, 17 mg of D-pantothenic acid, 21 mg of niacin, 1.9 mg of ethoxyquin, 112 mg of Fe (ferrous sulfate), 112 mg of Zn (zinc sulfate), 51 mg of Mn (manganous oxide), 20 mg of Cu (copper chloride), 0.78 mg of I (calcium iodate); and 0.17 mg of Se (sodium selenite).

Following 6 d of acclimation to individual pens, pigs were enrolled in 2 experimental periods (**P**). During P1 (2 d), pigs were housed in TN conditions (20.5 ± 1.5 °C; 47.1 ± 8.4% relative humidity) for the collection of baseline measurements. During P2 (24 h), HSCON and HSMitoQ pigs were exposed to constant HS (37.3 ± 1.3 °C; 27.6 ± 4.2% relative humidity), while TNCON and TNMitoQ remained in TN conditions. Both room temperature and humidity were monitored and recorded every 5 min by 3 data loggers (Lascar EL-USB-2LCD, Erie, PA), interspersed throughout each room.

Therapeutic treatments consisted of 10 g of cookie dough containing either no therapeutic enrichment (CON) or 20 mg MitoQ (40 mg/d; MitoQ, Auckland, New Zealand). Treatments were equally split and administered orally twice daily (~0700 and 1800 hours) during P1 and P2. The MitoQ dose regimen was adapted from earlier experiments in rodents and humans (Snow et al., 2010) and replicated from our previous study in similar sized barrows (Mayorga et al., 2024).

### Data collection

Rectal temperature (***T***_**R**_), skin temperature (***T***_**S**_), and respiration rate (**RR**) were obtained twice daily (~0700 h and 1800 hours) during P1 and at 0, 4, 8, 12, 16, 20, and 24 h during P2. Rectal temperature was measured using a calibrated electronic thermometer (SureTemp Plus 590, accuracy: ± 0.1 °C WelchAllyn, Skaneateles Falls, NY). Skin temperature was measured at the rump area using a calibrated infrared thermometer equidistant from the skin across pigs (Southwire digital thermometer, accuracy ± 2 °C, Southwire Company, LLC., Carrollton, GA). RR was determined by counting flank movements for 15 s and multiplying the observed rate by 4 to obtain breaths per minute (**bpm**). Feed intake (**FI**) was measured daily during P1 and at 4, 8, 12, 16, 20, and 24 h during P2 as feed disappearance. BWs were recorded at the end of the acclimation period, at the beginning of P2, and immediately prior to euthanasia. BW change was calculated by subtracting final BW from the BW recorded prior to P2.

Pigs were euthanized at the end of the 24 h challenge with the captive bolt technique followed by exsanguination. The entire gastrointestinal tract was removed and the stomach, small, and large intestine contents were removed and individually weighed. Weights of the empty (with contents removed) stomach, small, and large intestine were recorded. The liver was also collected and weighed.

### Blood sampling and analysis

Blood samples from the jugular vein were collected into a disposable tube (~3 mL, plasma, K_2_EDTA BD Vacutainer, Franklin Lakes, NJ) before exsanguination and later submitted to the Iowa State University Clinical Pathology Laboratory (Ames, IA) for automated-differential complete blood count analysis using a flow cytometry-based hematology analyzer (ADVIA 2120i; Siemens, Munich, Germany).

### Statistical analysis

Data were statistically analyzed using the MIXED procedure of SAS version 9.4 (SAS Inst. Inc., Cary, NC). Hourly measurements (i.e., body temperature indices and FI) were analyzed by repeated measures with an autoregressive covariance structure and time as the repeated factor. The model included environment (TN or HS), therapeutic (CON or MitoQ), time, and their interactions, and block as fixed effects; pig was used as the random effect. Each individual animals’ acclimation data were used as a covariate for *T*_R_, *T*_S_, and RR. Additionally, blood parameters, gastrointestinal measurements, and BW data were analyzed using PROC MIXED with a diagonal covariance structure. Data are reported as least squares means and considered significant if *P* ≤ 0.05 and a tendency if 0.05 < *P* ≤ 0.10.

## Results

### Body temperature indices and growth performance

**Figure 1.**
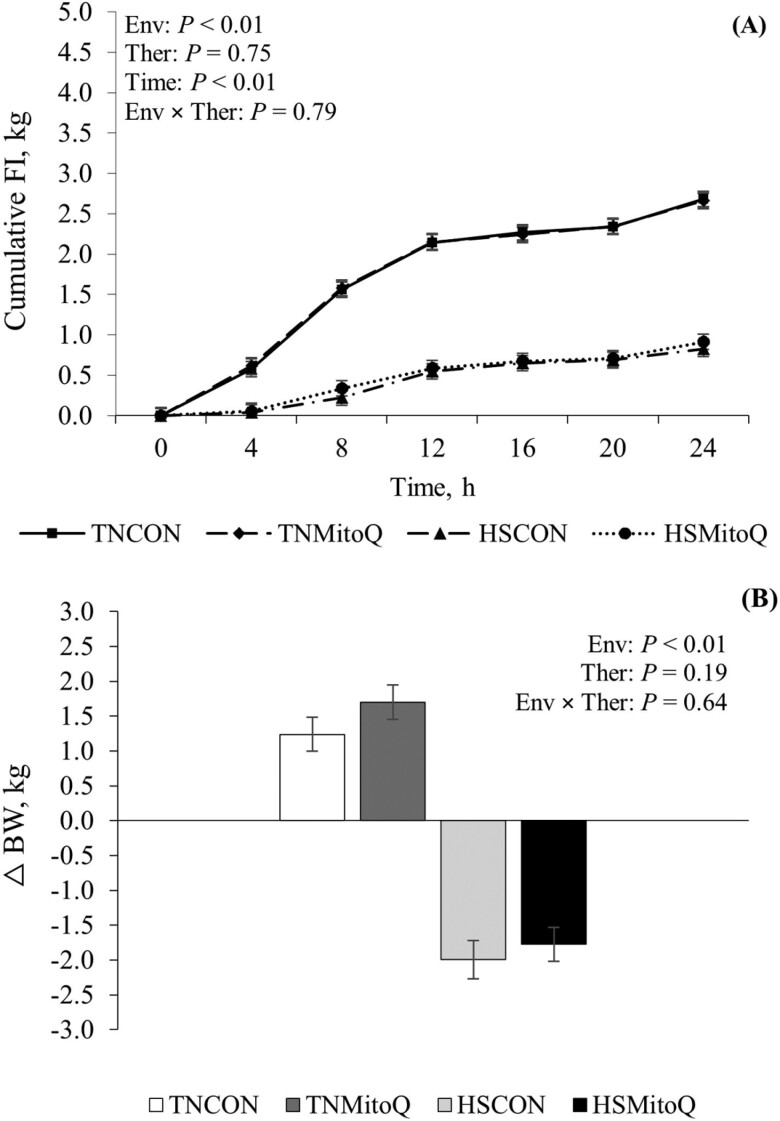
Effects of MitoQ on (A) cumulative FI and (B) change in BW (final—initial BW) during a 24-h acute HS challenge in growing gilts. Treatments: TNCON = thermoneutral control; TNMitoQ = thermoneutral MitoQ; HSCON = heat stress control; HSMitoQ = heat stress MitoQ. Env = environment. Ther = therapeutic. Data are represented as least squares means ± standard error of the mean and considered significant if *P* ≤ 0.05 and a tendency if 0.05 < *P* ≤ 0.10.

During HS, pigs had increased *T*_R_, *T*_S_, and RR compared to their TN counterparts. (1.5 °C, 8.7 °C, and 86 bpm, respectively; *P* < 0.01; Table 2). FI decreased (67%; *P *< 0.01; Table 2; Figure 1A) in HS pigs compared to TN pigs. Additionally, by the end of the 24 h HS challenge, HS pigs lost BW while TN pigs gained BW (−1.9 vs. +1.5 kg, respectively; *P* < 0.01; Table 2; Figure 1B). Supplementing MitoQ did not affect the aforesaid body temperature indices, FI, or BW loss (*P* ≥ 0.19).

**Table 2.** Effects of MitoQ on body temperature indices, FI, and delta BW during a 24-h acute HS challenge in growing gilts

Parameter	TN^1^		HS^2^		SEM	*P* value						
	CON^3^	MitoQ^4^	CON^3^	MitoQ^4^		Env^5^	Ther^6^	Time	Env × Ther^7^	Env × Time^8^	Ther × Time^9^	Env × Ther × Time^10^
T_R_^11^, °C	39.11	39.12	40.61	40.59	0.06	<0.01	0.91	<0.01	0.81	0.02	0.21	0.43
T_S_^12^, °C	35.68	35.94	45.03	44.81	0.32	<0.01	0.95	<0.01	0.44	<0.01	0.93	0.94
RR^13^, bpm	32	32	120	115	3.42	<0.01	0.49	0.01	0.46	0.17	0.48	0.73
Total FI^14^, kg	2.68	2.66	0.83	0.91	0.11	<0.01	0.76	—	0.64	—	—	—
Δ BW^15^, kg	1.24	1.70	-1.99	-1.78	0.27	<0.01	0.19	—	0.64	—	—	—

^1^Thermoneutral.

^2^Heat stress.

^3^Control.

^4^Mitoquinol.

^5^Environmental.

^6^Therapeutic.

^7^Environment by therapeutic interaction.

^8^Environment by time interaction.

^9^Therapeutic by time interaction.

^10^Environment by therapeutic by time interaction.

^11^Rectal temperature.

^12^Skin temperature.

^13^Respiration rate.

^14^Feed intake.

^15^Body weight change following a 24-h acute HS challenge.

### Gastrointestinal tract and liver weights

Overall, total GIT weight was decreased in HS relative to TN pigs (6.69 vs. 5.63 kg, respectively; *P* = 0.01; Table 3), and this was mainly driven by decreased luminal contents (2.43 vs. 3.26 kg, respectively; *P* = 0.01) and reduced empty GIT mass (3.21 vs. 3.48 kg, respectively; *P *= 0.03). Interestingly, stomach contents did not differ between HS and TN pigs (*P* > 0.54) but tended to be increased in MitoQ relative to CON pigs (0.89 vs. 0.62 kg, respectively; *P* = 0.08; Table 3). Conversely, small and large intestine contents were reduced (49% and 34%, respectively; *P* < 0.01; Table 3) in HS relative to TN pigs. To determine whether changes in gut peristalsis were specific to the proximal or distal regions of the GIT, luminal contents within each segment were expressed as a percentage of total GIT contents. Interestingly, the percentage of luminal contents remained similar across treatments in the stomach and large intestine (*P* > 0.15) but was decreased in the small intestine in HS pigs relative to their TN counterparts (14% vs. 21%; respectively; *P* < 0.01; Table 3). Due to the large differences in FI between HS and TN pigs, we further calculated total luminal contents as a percentage of the previous day’s FI and observed an overall increase in HS relative to TN pigs (350% vs. 124%; respectively; *P* < 0.01; Table 3). Within each GIT segment, luminal contents as a percentage of FI were increased in the stomach (93% vs. 31%; *P* < 0.01), small (47% vs. 26%; *P* < 0.01), and large intestine (210% vs. 67%; *P* < 0.01) in HS relative to TN pigs, respectively.

**Table 3.** Effects of MitoQ on gastrointestinal measurements following a 24-h acute HS challenge in growing gilts

Parameter	TN^1^		HS^2^		SEM	*P* value		
	CON^3^	MitoQ^4^	CON^3^	MitoQ^4^		Env^5^	Ther^6^	Env × Ther^7^
GIT weight^8^, kg	6.37	7.02	5.53	5.73	0.29	<0.01	0.16	0.46
Luminal contents^9^, kg	2.98	3.54	2.29	2.56	0.23	<0.01	0.08	0.55
Stomach	0.66	0.95	0.59	0.84	0.15	0.54	0.08	0.88
Small intestine	0.64	0.76	0.37	0.35	0.06	<0.01	0.36	0.24
Large intestine	1.68	1.82	1.32	1.37	0.12	<0.01	0.45	0.70
Luminal contents, %^10^								
Stomach	20.5	26.6	25.0	32.1	3.4	0.15	0.07	0.88
Small intestine	21.1	21.8	15.4	13.6	1.6	<0.01	0.73	0.43
Large intestine	58.4	51.6	59.5	54.2	3.7	0.61	0.12	0.84
Luminal contents, % FI^11^	112	136	360	341	52	<0.01	0.97	0.68
Stomach	24.4	37.7	84.0	101.9	12.0	<0.01	0.21	0.85
Small intestine	23.6	28.6	48.5	45.3	6.0	<0.01	0.88	0.50
Large intestine	64.5	69.8	227.7	193.4	38.2	<0.01	0.71	0.61
Empty tissue^12^, kg	3.48	3.49	3.24	3.17	0.12	0.03	0.79	0.75
Stomach	0.48	0.48	0.45	0.46	0.02	0.17	0.69	0.82
Small intestine	1.74	1.69	1.56	1.57	0.06	0.02	0.77	0.66
Large intestine	1.27	1.31	1.23	1.14	0.08	0.18	0.77	0.38
Empty tissue, % BW^13^	5.46	5.45	5.07	4.97	0.20	0.04	0.79	0.83
Stomach	0.75	0.75	0.70	0.72	0.03	0.23	0.69	0.77
Small intestine	2.72	2.65	2.44	2.47	0.09	0.02	0.84	0.59
Large intestine	1.99	2.05	1.93	1.78	0.12	0.21	0.71	0.41
Liver weight, kg	1.28	1.16	1.20	1.11	0.03	0.02	<0.01	0.55
Liver, % BW^14^	2.01	1.81	1.88	1.75	0.05	0.05	<0.01	0.49

^1^Thermoneutral.

^2^Heat stress.

^3^Control.

^4^Mitoquinol.

^5^Environment.

^6^Therapeutic.

^7^Environment by therapeutic interaction.

^8^Total gastrointestinal tract weight (contents + empty tissue from the stomach and small and large intestines).

^9^Total gastrointestinal tract contents (stomach + small and large intestines).

^10^Luminal contents expressed as a percentage of total gastrointestinal tract contents.

^11^Luminal contents expressed as a percentage of total FI during a 24-h acute HS challenge.

^12^Total empty gastrointestinal tract weight (stomach + small and large intestines weights without contents).

^13^Total empty gastrointestinal tract expressed as a percentage of final BW.

^14^Liver weight expressed as a percentage of final BW.

Empty GIT weight was decreased in HS relative to TN pigs (3.20 vs. 3.48 kg, respectively; *P* = 0.03; Table 3), and this was mainly driven by decreased empty small intestine in HS compared to TN pigs (1.56 vs. 1.71 kg, respectively; *P* = 0.02) as empty stomach and large intestine weights remained similar across environments and therapeutic treatments (*P* > 0.17; Table 3). Following this same trend, empty GIT weight, as percentage of BW, was decreased in HS relative to TN pigs (5.02% vs. 5.45%, respectively; *P* = 0.04; Table 3), and this was influenced by reduced small intestine weight in HS relative to their TN counterparts (2.45% vs. 2.68%; *P* = 0.02) as stomach and large intestine weights, as percentage of BW, remained similar across treatments (*P* > 0.21; Table 3).

Liver weight was decreased in HS compared to TN pigs (1.15 vs. 1.22 kg, respectively; *P* = 0.02; Table 3), and it was further decreased in MitoQ compared to CON pigs (1.13 vs. 1.24 kg, respectively; *P* < 0.01; Table 3). Similarly, liver weight as a percentage of BW was decreased in HS relative to TN pigs (1.81% vs. 1.91%, respectively; *P* = 0.05) and was further decreased in pigs fed MitoQ relative to their CON counterparts (1.78% vs. 1.94%, respectively; *P* < 0.01).

### Blood hematology

In general, no differences were observed on any hematologic parameter across environment or therapeutic treatments (*P* > 0.16; Table 4); however, circulating lymphocytes tended to decrease in HS compared to TN pigs (16%; *P* = 0.07; Table 4).

**Table 4.** Effects of MitoQ on hematology parameters following a 24-h acute HS challenge in growing gilts

Parameter	TN^1^		HS^2^		SEM	*P* value		
	CON^3^	MitoQ^4^	CON^3^	MitoQ^4^		Env^5^	Ther^6^	Env × Ther^7^
WBC^8^, × 10^3^/μL	26.58	24.79	23.28	22.71	1.54	0.16	0.49	0.69
Neutrophils, × 10^3^/μL	6.71	6.14	5.99	5.63	0.40	0.25	0.33	0.78
Lymph.^9^, × 10^3^/μL	17.55	16.93	14.25	14.76	1.21	0.07	0.97	0.71
Monocytes, × 10^3^/μL	1.40	1.53	1.21	1.28	0.72	0.19	0.38	0.92
Eosinophils, × 10^3^/μL	0.44	0.48	0.45	0.47	0.16	0.99	0.82	0.93
Basophils, × 10^3^/μL	0.32	0.26	0.22	0.15	0.07	0.24	0.44	0.99
RBC^10^, × 10^6^/μL	7.37	7.50	7.30	7.11	0.33	0.51	0.95	0.60
Hemoglobin, g/dL	13.54	13.40	13.55	12.90	0.55	0.69	0.55	0.61
Hematocrit, %	45.03	42.97	43.51	40.95	1.93	0.44	0.35	0.88

^1^Thermoneutral.

^2^Heat Stress.

^3^Control.

^4^Mitoquinol.

^5^Environment.

^6^Therapeutic.

^7^Environment by therapeutic interaction.

^8^White blood cells.

^9^Lymphocytes.

^10^Red blood cells.

## Discussion

HS is an economic burden to animal agriculture due to its adverse effects on animal welfare and productivity (Key and Sneeringer, 2014). Production losses due to HS are mainly associated with reduced and inconsistent growth rates, decreased meat and milk production, reduced reproductive performance, and increased mortality and morbidity (Renaudeau et al., 2012; Baumgard and Rhoads, 2013). The exact mechanism underlying these physiological changes during HS remains elusive. However, compelling evidence suggests that decreased intestinal barrier function, ostensibly due, at least in part, to HS-induced OS, is likely the origin of many adverse effects of HS (Pearce et al., 2013; Gabler and Pearce, 2015; Gabler et al., 2018; Lim, 2018; Koch et al., 2019; Lian et al., 2020; Mayorga et al., 2020; Rostagno, 2020; Ortega and Szabó, 2021). Therefore, identifying mitigation strategies to ameliorate intestinal barrier dysfunction during HS is critical to minimize production losses and improve animal welfare during the warm summer months. Thus, study objectives were to assess the effects of MitoQ on key production metrics and gross variables of GIT mass during an acute HS challenge in growing gilts.

Pigs exposed to acute HS had marked increases in T_R_, T_S_, and RR; metrics that demonstrate we successfully implemented a severe heat load. However, supplementing MitoQ did not appear to affect body temperature indices, corroborating our earlier experiment (Mayorga et al., 2024). As anticipated, pigs exposed to HS markedly decreased FI (67%) compared to TN pigs, and this magnitude was consistent with our previous HS studies (Mayorga et al., 2021, 2024). Reduced voluntary FI is a conserved response during HS, and a survival mechanism to minimize metabolic heat production (Quiniou et al., 2000). Contrary to our expectations, MitoQ administration did not affect FI during HS, which disagrees with our previous experiment, wherein MitoQ-fed barrows had increased FI compared to HS controls (Mayorga et al., 2024). The effects of MitoQ on FI have not been extensively evaluated, particularly in pigs. Supplemented to male rodents receiving a high-fat diet, MitoQ appears to reduce FI; however, MitoQ did not affect FI when supplemented to male rodents receiving a typical (non-high-fat) diet (Fink et al., 2017). Further, heat-stressed pigs lost a considerable amount of BW (~2.0 kg), but MitoQ administration did not influence this response. This also disagrees with our earlier findings, wherein MitoQ attenuated the severity of BW loss in heat-stressed barrows (Mayorga et al., 2024). Reasons MitoQ did not improve FI and BW, similarly to our previous experiment are unclear as the MitoQ manufacturer and dose were identical, and the size of the pigs used in both studies was similar. Although the heat load applied in the current study differed slightly from our previous experiment (37 vs. 35 °C, respectively), the body temperature and FI responses herein were comparable to our previous experiment (Mayorga et al., 2024). One obvious and intentional difference between experiments is biological sex, and some evidence suggests HS may have a more detrimental effect on females (Kenney, 1985; Lublin et al., 1995; Rudolph et al., 2021). Because females were employed in this experiment, it is plausible that the heat load and the subsequent OS were too severe for MitoQ to express its anticipated benefits. While it appears that males have increased ROS production and reduced antioxidant capacity compared to females (Martinez de Toda et al., 2023), making comparisons between studies is fraught with potential confounding issues, not least of which include sex steroids. Furthermore, although the diets utilized in the current and previous experiments (Mayorga et al., 2024) were intended to be similar, potential variations in the biological activity of dietary antioxidants could have contributed to the observed discrepancies. Regardless, there were clear distinctions in response to MitoQ supplementation during HS in these investigations (affecting both FI and BW), and identifying the underlying mechanism contributing to this knowledge gap has pragmatic and economic implications for the swine industry.

**Figure 2.**
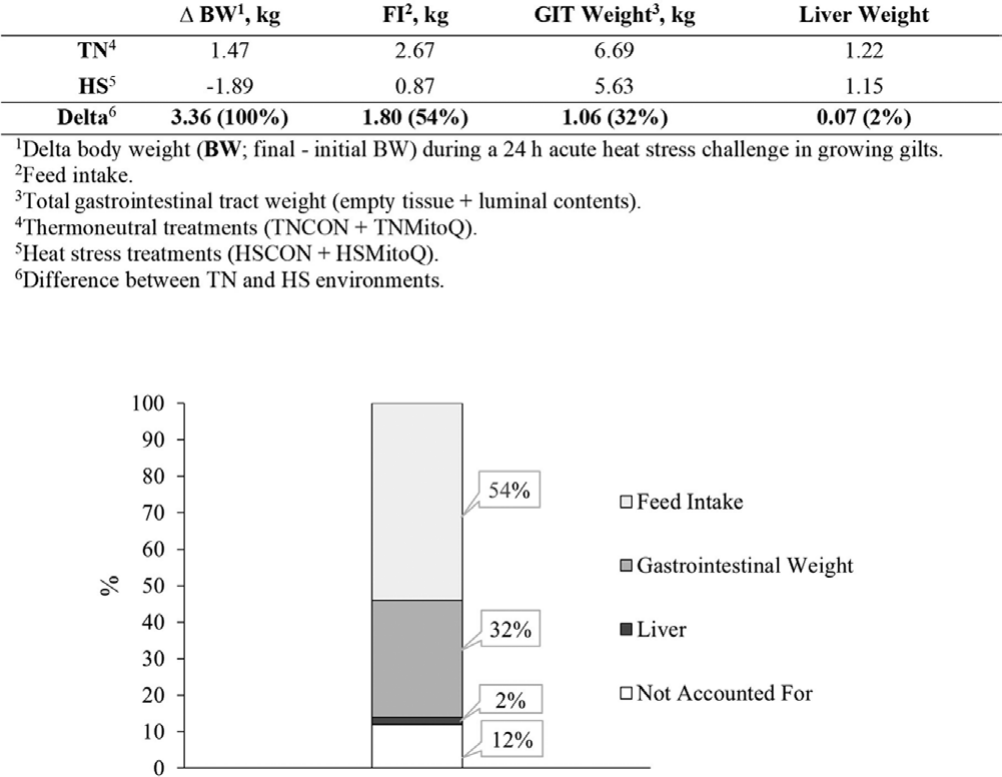
Effects of HS on BW loss during a 24-h acute HS challenge in growing gilts. The impact of reduced FI and decreased gastrointestinal tract and liver weight was calculated and illustrated as percentage of total BW loss.

Reduced FI (67%) only partially explains the reduction in BW (~2 kg) following 24 h of acute HS. The splanchnic bed mass (i.e., GIT, liver, spleen) is reduced after chronic HS exposure (Johnson et al., 2015; Cui et al., 2016); however, it remains unclear if acute HS has similar effects. Herein, heat-stressed pigs had reduced overall GIT mass compared to TN pigs, which was explained by both decreased empty GIT mass and luminal contents. We have previously reported a similar decrease in GIT weight in heat-stressed pigs relative to their TN counterparts following a 24-h acute HS challenge and after a 7 d HS challenge in growing barrows and gilts (Mayorga et al., 2024; Rudolph et al., 2024). Interestingly, irrespective of environment, MitoQ pigs had a numerical increase (*P *> 0.16) in total tissue weight (tissue and luminal contents) relative to CON, which contradicts our previous study. In addition to reduced GIT mass, the thermoregulatory mechanisms employed during HS (i.e., panting) might have increased the loss of body fluids, potentially contributing to the reduction in BW, as observed in human studies (Akerman et al., 2016). However, in this study, hematocrit did not differ between environmental groups; therefore, it is unlikely that dehydration had a meaningful impact on the observed decrease in BW. In summary, while reduced BW following HS is partially explained by decreased FI, and reduced GIT and liver weights, the factors accounting for the remaining portion (0.43 kg; Figure 2) remain unclear. Further research is warranted to understand the physiological mechanisms underlying the observed BW loss in response to acute HS.

Corticotropin-releasing factor (**CRF**) may play a role in stress-induced alterations in GIT physiology (Taché et al., 1999) and could partially explain the reduction in BW after HS. Numerous studies have indicated that CRF partially controls gut motility by delaying gastric emptying and increasing small intestinal transit (Czimmer and Taché, 2017); however, its effects during acute HS remain unknown. We previously reported an increased percentage of luminal contents in heat-stressed barrows after a 24-h thermal event, regardless of decreased FI, indicating HS altered gut peristalsis, likely by reducing GIT motility (Mayorga et al., 2024; Rudolph et al., 2024). However, our previous study was limited as measurements did not distinguish between GIT segments, so we were unable to determine whether alterations in gut peristalsis were specific to the proximal or distal regions of the GIT (Mayorga et al., 2024). In the present study, while intestinal luminal contents were decreased in heat-stressed pigs, stomach contents remained similar across treatments. When luminal contents were expressed as a percentage of total contents, it seems heat-stressed pigs retain a comparable amount of luminal contents in the stomach and large intestine than TN pigs. This observation is especially remarkable because FI was drastically reduced in heat-stressed pigs. Thus, to account for dissimilar FI, we further expressed luminal contents as a percentage of FI and observed a substantial increase in luminal contents in all segments of the GIT in heat-stressed compared to TN pigs, with the stomach and large intestine disproportionately contributing to this phenomenon. A limitation to our interpretation is that we did not determine digesta dry matter and thus luminal contents likely consisted of more water because they ostensibly drank more. Regardless of the governing mechanism(s), the important question is, “Why are there clear segment differences in motility?”. Notably, HS appears to slow gastric emptying while simultaneously increasing intestinal transit. We speculate that it is a strategy to minimize intestinal luminal content and pathogen load, thereby reducing the antigen burden that could potentially infiltrate the epithelial barrier. Understanding what controls GIT motility and its contribution to suboptimal growth during HS is crucial to developing therapeutic interventions to minimize production losses in farm animals and potentially improve health outcomes for humans experiencing heat-related illnesses and heat stroke.

Interestingly, MitoQ reduced overall liver size regardless of environment. Reasons why are not clear; however, MitoQ has been previously shown to attenuate the increase in liver size in some metabolic and hepatic disease rodent models (Coudray et al., 2016; Turkseven et al., 2020), but conflicting results have been observed in others (Junior et al., 2018; Gal et al., 2021). The mechanisms underlying this response to MitoQ administration could include a reduction in hepatic inflammation (Turkseven et al., 2020) or liver lipid content (Coudray et al., 2016), which may be particularly relevant in the context of HS. Regardless, understanding why MitoQ decreases liver size is of interest, given the liver’s pivotal role as the master regulator of intermediary metabolism and its economic implications for animal agriculture.

## Conclusion

The current experiment evaluated the therapeutic effects of MitoQ during a 24-h acute HS challenge in growing gilts. As anticipated, pigs exposed to HS had increased body temperature indices and a drastic decrease in FI and BW. Furthermore, liver weight, GIT mass, and luminal contents were decreased in heat-stressed pigs. Intriguingly, it appears HS reduces gastric emptying while simultaneously increasing motility in the small and large intestines. Although the reasons for these observations are unclear, having a better understanding will likely provide foundational information necessary to develop preventative and therapeutic strategies during HS. Contrary to our initial hypothesis, MitoQ supplementation during HS had limited to no effect on variables measured herein, which disagrees with our previous MitoQ study in growing barrows. Whether these differences are attributed to biological sex remains unknown but deserves further investigation as it would open opportunities for a more targeted approach to mitigate the negative effects of hyperthermia on production farm animals. Future research should also explore whether ameliorating OS via antioxidant supplementation other than MitoQ enhances porcine performance and health during HS.

## Abbreviations

bpm: breaths per minute
BW: body weight
CON: control
CRF: corticotrophin releasing factor
FI: feed intake
GIT: gastrointestinal tract
HS: heat stress
HSCon: heat stress + control
HSMitoQ: heat stress + mitoquinol
MitoQ: mitoquinol
OS: oxidative stress
P: period
ROS: reactive oxygen species
RR: respiration rate
TN: thermoneutral
TNCon: thermoneutral + control
TNMitoQ: thermoneutral + mitoquinol
*T*_R_: rectal temperature
*T*_S_: skin temperature
